# Supplementary material for: Hybrid Models and Biological Model Reduction with PyDSTool
Source: PLoS Comput Biol. 2012 Aug 9;8(8):e1002628. doi: 10.1371/journal.pcbi.1002628 (PMC3415397; doi:10.1371/journal.pcbi.1002628)
Supplement: Text S4 — Complete source code for the PyDSTool package (version 0.88.120504). Includes API documentation and help files linking to web pages. This file is identical to the current public release on Sourceforge.net. (ZIP) [file pcbi.1002628.s004.zip › PyDSTool/html/PyDSTool.Toolbox.NineML-module.html]

xml version="1.0" encoding="ascii"?


PyDSTool.Toolbox.NineML


| Home | Trees | Indices | Help | PyDSTool |
| --- | --- | --- | --- | --- |

Package PyDSTool ::
Package Toolbox ::
Module NineML

[hide private]

[frames] | no frames]

# Module NineML

source code

PySCes interface code for systems biology modeling and SBML model
markup.

This toolbox assumes you have PySCes installed.

R. Clewley, 2012


|  |  |
| --- | --- |
| Classes | [hide private] |

NineMLModel


|  |  |
| --- | --- |
| Functions | [hide private] |

|  |  |
| --- | --- |
| get\_nineml\_model(c, model\_name, target=`'``Vode``'`, extra\_args=None, alg\_args=None, max\_t=inf)  component is a NineML abstraction layer component, and assumes you have imported a NineML model through the Python API or built it directly. | source code |

|  |  |
| --- | --- |
| get\_regime\_model(r, fns, sigs) | source code |


|  |  |
| --- | --- |
| Variables | [hide private] |

acosh

replaceSepStr

newton\_meth

norm

float96

protected\_randomnames

ONES

typeCounter

specialfns

ensuredecimalconst

toCircumflexSyntax

mathlookup

Continuous = `Continuous Domain`

toPowSyntax

rerr = `<PyDSTool.Redirector.Redirector object at 0x4d185b0>`

sym2name

sinh = `<ufunc 'sinh'>`

getlftrtD

doadd

less\_equal = `<ufunc 'less_equal'>`

replaceCallsWithDummies

randomlookup

cPickle

replaceSepQSpec

feval\_map\_const

protected\_auxnamesDB = `ModelSpec internal helper class: auxfnD...`

Inf = `inf`

specTypes = `('RHSfuncSpec', 'ImpFuncSpec', 'ExpFuncSpec')`

MReg

logical\_or = `<ufunc 'logical_or'>`

errorfields = `{0: ['t', 'error info'], 10: ['t', 'event list']}`

tan = `<ufunc 'tan'>`

math\_dir

atanh

name\_chars\_RE

temp\_macro\_names\_inv

arccos = `<ufunc 'arccos'>`

isVectorClause

POW\_STR

convert\_power\_reserved\_keywords

W\_NONTERMSTATEBD = `21`

predicate\_op

cosh = `<ufunc 'cosh'>`

dsInterface

inverseMathNameMap = `{'Random': 'Random', 'SystemRandom': 'Sys...`

degrees = `<ufunc 'degrees'>`

greater = `<ufunc 'greater'>`

E\_NONUNIQUETERM = `10`

radians = `<ufunc 'radians'>`

sin = `<ufunc 'sin'>`

symbolMapClass

token

API = `API_class()`

fmod = `<ufunc 'fmod'>`

ensureints

API\_class

funcnames

mathNameMap = `{'Abs': 'abs', 'Acos': 'acos', 'Asin': 'asin', '...`

alphabet\_chars\_RE

re

contained = `contained`

toDoubleStarSyntax

protected\_allnames = `['acos', 'asin', 'atan', 'atan2', 'ceil',...`

ensureparen

traceback

math\_globals

ZEROS

reserved\_keywords

arctan2 = `<ufunc 'arctan2'>`

ldexp = `<ufunc 'ldexp'>`

mapNames

trysimple

replaceSep

complex192

temp\_macro\_names

arctanh = `<ufunc 'arctanh'>`

string2ast

W\_UNCERTVAL = `0`

null\_predicate\_class

rout = `<PyDSTool.Redirector.Redirector object at 0x4d18cb0>`

dodiv

parserObject

simplify

minpack

feature\_node

isNameToken

W\_TERMSTATEBD = `11`

fit\_function

exp = `<ufunc 'exp'>`

DO\_POW

nameResolver = `ModelSpec internal helper class: nameResolver o...`

DomainType

findEndBrace

frexp = `<ufunc 'frexp'>`

simplify\_str

mapPowStr

ceil = `<ufunc 'ceil'>`

builtin\_auxnames

ensurebare

regObject

targetLangs = `['c', 'python', 'matlab']`

allmathnames = `['acos', 'asin', 'atan', 'atan2', 'ceil', 'cos'...`

isnan = `<ufunc 'isnan'>`

strIfSeq

feval\_map\_symb

isIntegerToken

protected\_specialfns

cos = `<ufunc 'cos'>`

theGenSpecHelper = `GenSpecHelper()`

feature\_leaf

whoQ

dofun

replaceSepListInv

HAVE\_PSYCO = `True`

replaceSepInv

protected\_mathnames

utils\_info

pi = `3.14159265359`

asinh

arcsin = `<ufunc 'arcsin'>`

doneg

accrueCompTypes

count\_sep

uncertain = `uncertain`

protected\_macronames

tanh = `<ufunc 'tanh'>`

DO\_DEC

builtinlookup

getdim

fabs = `<ufunc 'fabs'>`

NaN = `nan`

sqrt = `<ufunc 'sqrt'>`

complex

ensureparen\_div

LargestInt32 = `2147483647`

allCompTypes

splitargs

splitastLR

dopower

TENS

processMultiDef

W\_NONTERMEVENT = `20`

joinStrs

qtypes

less = `<ufunc 'less'>`

W\_TERMEVENT = `10`

verstr\_parts = `['0', '7', '0']`

errmessages = `{0: 'Computation of trajectory failed', 10: 'Mor...`

process\_raw\_residual

W\_BISECTLIMIT = `12`

isNumericToken

isHierarchicalName

genDB = `Generator internal database class: []`

mod = `<ufunc 'remainder'>`

builtinFnSigInfo

warnfields = `{0: ['value', 'interval'], 10: ['t', 'event list'...`

Macheps

hypot = `<ufunc 'hypot'>`

ast2string

domul

remove\_indices\_from\_range

null\_predicate = `null_predicate_class(None)`

collect\_numbers

modf = `<ufunc 'modf'>`

power = `<ufunc 'power'>`

syms

Discrete = `Discrete Domain`

findNumTailPos

makeParList

num\_chars

readArgs

notcontained = `notcontained`

symbolMapDict = `{}`

resolveSpecTypeCombos

arcsinh = `<ufunc 'arcsinh'>`

joinAsStrs

proper\_match

log = `<ufunc 'log'>`

allmathnames\_symbolic = `['Acos', 'Asin', 'Atan', 'Atan2', 'Cei...`

log10 = `<ufunc 'log10'>`

ast2shortlist

modlookup

isToken

replaceSepList

dosub

arccosh = `<ufunc 'arccosh'>`

parser

E\_COMPUTFAIL = `0`

parseMatrixStrToDictStr

warnmessages = `{0: 'Uncertain value computed', 10: 'Terminal e...`

floor = `<ufunc 'floor'>`

arctan = `<ufunc 'arctan'>`

local\_fndef

protected\_scipynames

symbol

e = `2.71828182846`

NAMESEP

\_seq\_types = `(<type 'list'>, <type 'tuple'>, <type 'numpy.ndar...`

\_num\_types = `(<type 'float'>, <type 'int'>, <type 'numpy.float...`

isfinite = `<ufunc 'isfinite'>`

sign = `<ufunc 'sign'>`

allODEgens = `['Radau_ODEsystem', 'ADMC_ODEsystem', 'Vode_ODEsy...`

\_functions = `['get_nineml_model']`

\_classes = `['NineMLModel']`

\_features = `[]`

ALLOW\_THREADS = `1`

Abs = `Abs (ModelSpec wrapper)`

Acos = `Acos (ModelSpec wrapper)`

Asin = `Asin (ModelSpec wrapper)`

Atan = `Atan (ModelSpec wrapper)`

Atan2 = `Atan2 (ModelSpec wrapper)`

BUFSIZE = `10000`

Betavariate = `Betavariate (ModelSpec wrapper)`

CLIP = `0`

Ceil = `Ceil (ModelSpec wrapper)`

Choice = `Choice (ModelSpec wrapper)`

Cos = `Cos (ModelSpec wrapper)`

Cosh = `Cosh (ModelSpec wrapper)`

Degrees = `Degrees (ModelSpec wrapper)`

E = `QuantSpec e (ExpFuncSpec)`

ERR\_CALL = `3`

ERR\_DEFAULT = `0`

ERR\_DEFAULT2 = `2084`

ERR\_IGNORE = `0`

ERR\_LOG = `5`

ERR\_PRINT = `4`

ERR\_RAISE = `2`

ERR\_WARN = `1`

Exp = `Exp (ModelSpec wrapper)`

Expovariate = `Expovariate (ModelSpec wrapper)`

FLOATING\_POINT\_SUPPORT = `1`

FPE\_DIVIDEBYZERO = `1`

FPE\_INVALID = `8`

FPE\_OVERFLOW = `2`

FPE\_UNDERFLOW = `4`

Fabs = `Fabs (ModelSpec wrapper)`

False\_ = `False`

Floor = `Floor (ModelSpec wrapper)`

Fmod = `Fmod (ModelSpec wrapper)`

Frexp = `Frexp (ModelSpec wrapper)`

Gammavariate = `Gammavariate (ModelSpec wrapper)`

Gauss = `Gauss (ModelSpec wrapper)`

Getrandbits = `Getrandbits (ModelSpec wrapper)`

Getstate = `Getstate (ModelSpec wrapper)`

Hypot = `Hypot (ModelSpec wrapper)`

Infinity = `inf`

Jumpahead = `Jumpahead (ModelSpec wrapper)`

Ldexp = `Ldexp (ModelSpec wrapper)`

Log = `Log (ModelSpec wrapper)`

Log10 = `Log10 (ModelSpec wrapper)`

Lognormvariate = `Lognormvariate (ModelSpec wrapper)`

MAXDIMS = `32`

Max = `Max (ModelSpec wrapper)`

Min = `Min (ModelSpec wrapper)`

Modf = `Modf (ModelSpec wrapper)`

NAN = `nan`

NINF = `-inf`

NZERO = `-0.0`

Normalvariate = `Normalvariate (ModelSpec wrapper)`

PINF = `inf`

PZERO = `0.0`

Paretovariate = `Paretovariate (ModelSpec wrapper)`

Pi = `QuantSpec pi (ExpFuncSpec)`

Pow = `Pow (ModelSpec wrapper)`

RAISE = `2`

Radians = `Radians (ModelSpec wrapper)`

Randint = `Randint (ModelSpec wrapper)`

Random = `Random (ModelSpec wrapper)`

Randrange = `Randrange (ModelSpec wrapper)`

SHIFT\_DIVIDEBYZERO = `0`

SHIFT\_INVALID = `9`

SHIFT\_OVERFLOW = `3`

SHIFT\_UNDERFLOW = `6`

Sample = `Sample (ModelSpec wrapper)`

ScalarType = `(<type 'int'>, <type 'float'>, <type 'complex'>, ...`

Seed = `Seed (ModelSpec wrapper)`

Setstate = `Setstate (ModelSpec wrapper)`

Shuffle = `Shuffle (ModelSpec wrapper)`

Sin = `Sin (ModelSpec wrapper)`

Sinh = `Sinh (ModelSpec wrapper)`

Sqrt = `Sqrt (ModelSpec wrapper)`

Sum = `Sum (ModelSpec wrapper)`

Systemrandom = `Systemrandom (ModelSpec wrapper)`

Tan = `Tan (ModelSpec wrapper)`

Tanh = `Tanh (ModelSpec wrapper)`

True\_ = `True`

UFUNC\_BUFSIZE\_DEFAULT = `10000`

UFUNC\_PYVALS\_NAME = `'UFUNC_PYVALS'`

Uniform = `Uniform (ModelSpec wrapper)`

Vonmisesvariate = `Vonmisesvariate (ModelSpec wrapper)`

WRAP = `1`

Weibullvariate = `Weibullvariate (ModelSpec wrapper)`

Wichmannhill = `Wichmannhill (ModelSpec wrapper)`

absolute = `<ufunc 'absolute'>`

add = `<ufunc 'add'>`

bitwise\_and = `<ufunc 'bitwise_and'>`

bitwise\_not = `<ufunc 'invert'>`

bitwise\_or = `<ufunc 'bitwise_or'>`

bitwise\_xor = `<ufunc 'bitwise_xor'>`

c\_ = `<numpy.lib.index_tricks.CClass object at 0x1154330>`

cast = `{<type 'numpy.int64'>: <function <lambda> at 0x10a8bb0>...`

conj = `<ufunc 'conjugate'>`

conjugate = `<ufunc 'conjugate'>`

copysign = `<ufunc 'copysign'>`

deg2rad = `<ufunc 'deg2rad'>`

divide = `<ufunc 'divide'>`

equal = `<ufunc 'equal'>`

exp2 = `<ufunc 'exp2'>`

expm1 = `<ufunc 'expm1'>`

floor\_divide = `<ufunc 'floor_divide'>`

fmax = `<ufunc 'fmax'>`

fmin = `<ufunc 'fmin'>`

greater\_equal = `<ufunc 'greater_equal'>`

index\_exp = `<numpy.lib.index_tricks.IndexExpression object at ...`

inf = `inf`

infty = `inf`

invert = `<ufunc 'invert'>`

isinf = `<ufunc 'isinf'>`

left\_shift = `<ufunc 'left_shift'>`

little\_endian = `True`

log1p = `<ufunc 'log1p'>`

logaddexp = `<ufunc 'logaddexp'>`

logaddexp2 = `<ufunc 'logaddexp2'>`

logical\_and = `<ufunc 'logical_and'>`

logical\_not = `<ufunc 'logical_not'>`

logical\_xor = `<ufunc 'logical_xor'>`

maximum = `<ufunc 'maximum'>`

mgrid = `<numpy.lib.index_tricks.nd_grid object at 0x113d490>`

minimum = `<ufunc 'minimum'>`

multiply = `<ufunc 'multiply'>`

n = `9`

nan = `nan`

nbytes = `{<type 'numpy.int64'>: 8, <type 'numpy.int16'>: 2, <t...`

negative = `<ufunc 'negative'>`

newaxis = `None`

nextafter = `<ufunc 'nextafter'>`

not\_equal = `<ufunc 'not_equal'>`

ogrid = `<numpy.lib.index_tricks.nd_grid object at 0x113dbb0>`

ones\_like = `<ufunc 'ones_like'>`

r\_ = `<numpy.lib.index_tricks.RClass object at 0x11542f0>`

rad2deg = `<ufunc 'rad2deg'>`

reciprocal = `<ufunc 'reciprocal'>`

remainder = `<ufunc 'remainder'>`

right\_shift = `<ufunc 'right_shift'>`

rint = `<ufunc 'rint'>`

s\_ = `<numpy.lib.index_tricks.IndexExpression object at 0x11543f0>`

sctypeDict = `{0: <type 'numpy.bool_'>, 1: <type 'numpy.int8'>,...`

sctypeNA = `{'?': 'Bool', 'B': 'UInt8', 'Bool': <type 'numpy.bo...`

sctypes = `{'complex': [<type 'numpy.complex64'>, <type 'numpy....`

signbit = `<ufunc 'signbit'>`

spacing = `<ufunc 'spacing'>`

square = `<ufunc 'square'>`

subtract = `<ufunc 'subtract'>`

t = `'0'`

true\_divide = `<ufunc 'true_divide'>`

trunc = `<ufunc 'trunc'>`

typeDict = `{0: <type 'numpy.bool_'>, 1: <type 'numpy.int8'>, 2...`

typeNA = `{'?': 'Bool', 'B': 'UInt8', 'Bool': <type 'numpy.bool...`

typecodes = `{'All': '?bhilqpBHILQPfdgFDGSUVOMm', 'AllFloat': '...`


|  |  |
| --- | --- |
| Function Details | [hide private] |

|  |  |
| --- | --- |
| get\_nineml\_model(c, model\_name, target=`'``Vode``'`, extra\_args=None, alg\_args=None, max\_t=inf) | source code |

component is a NineML abstraction layer component, and assumes you
have imported a NineML model through the Python API or built it
directly.

Currently only works for 'flat' models

  


|  |  |
| --- | --- |
| Variables Details | [hide private] |

### protected\_auxnamesDB

Value:
:   ```
    ModelSpec internal helper class: auxfnDBclass object
    ```

### inverseMathNameMap

Value:
:   ```
    {'Random': 'Random',
     'SystemRandom': 'Systemrandom',
     'WichmannHill': 'Wichmannhill',
     'abs': 'Abs',
     'acos': 'Acos',
     'asin': 'Asin',
     'atan': 'Atan',
     'atan2': 'Atan2',
    ...
    ```

### mathNameMap

Value:
:   ```
    {'Abs': 'abs',
     'Acos': 'acos',
     'Asin': 'asin',
     'Atan': 'atan',
     'Atan2': 'atan2',
     'Betavariate': 'betavariate',
     'Ceil': 'ceil',
     'Choice': 'choice',
    ...
    ```

### protected\_allnames

Value:
:   ```
    ['acos',
     'asin',
     'atan',
     'atan2',
     'ceil',
     'cos',
     'cosh',
     'degrees',
    ...
    ```

### nameResolver

Value:
:   ```
    ModelSpec internal helper class: nameResolver object
    ```

### allmathnames

Value:
:   ```
    ['acos',
     'asin',
     'atan',
     'atan2',
     'ceil',
     'cos',
     'cosh',
     'degrees',
    ...
    ```

### errmessages

Value:
:   ```
    {0: 'Computation of trajectory failed',
     10: 'More than one terminal event found'}
    ```

### warnfields

Value:
:   ```
    {0: ['value', 'interval'],
     10: ['t', 'event list'],
     11: ['t', 'var name', 'var value', '\n\tvalue interval'],
     12: ['t', 'event list'],
     20: ['t', 'event list'],
     21: ['t', 'var name', 'var value', '\n\tvalue interval']}
    ```

### allmathnames\_symbolic

Value:
:   ```
    ['Acos',
     'Asin',
     'Atan',
     'Atan2',
     'Ceil',
     'Cos',
     'Cosh',
     'Degrees',
    ...
    ```

### warnmessages

Value:
:   ```
    {0: 'Uncertain value computed',
     10: 'Terminal event(s) found',
     11: 'State variable reached bounds (terminal)',
     12: 'Bisection limit reached for event',
     20: 'Non-terminal event(s) found',
     21: 'State or input variable reached bounds (non-terminal)'}
    ```

### \_seq\_types

Value:
:   ```
    (<type 'list'>, <type 'tuple'>, <type 'numpy.ndarray'>)
    ```

### \_num\_types

Value:
:   ```
    (<type 'float'>,
     <type 'int'>,
     <type 'numpy.floating'>,
     <type 'numpy.integer'>)
    ```

### allODEgens

Value:
:   ```
    ['Radau_ODEsystem',
     'ADMC_ODEsystem',
     'Vode_ODEsystem',
     'Euler_ODEsystem',
     'Dopri_ODEsystem']
    ```

### ScalarType

Value:
:   ```
    (<type 'int'>,
     <type 'float'>,
     <type 'complex'>,
     <type 'long'>,
     <type 'bool'>,
     <type 'str'>,
     <type 'unicode'>,
     <type 'buffer'>,
    ...
    ```

### cast

Value:
:   ```
    {<type 'numpy.int64'>: <function <lambda> at 0x10a8bb0>, <type 'numpy.
    int16'>: <function <lambda> at 0x10a8bf0>, <type 'numpy.complex128'>: 
    <function <lambda> at 0x10a8c30>, <type 'numpy.uint64'>: <function <la
    mbda> at 0x10a8c70>, <type 'numpy.complex256'>: <function <lambda> at 
    0x10a8cf0>, <type 'numpy.float32'>: <function <lambda> at 0x10a8d30>, 
    <type 'numpy.bool_'>: <function <lambda> at 0x10a8cb0>, <type 'numpy.u
    int8'>: <function <lambda> at 0x10a8db0>, <type 'numpy.int32'>: <funct
    ion <lambda> at 0x10a8eb0>, <type 'numpy.int8'>: <function <lambda> at
    ...
    ```

### index\_exp

Value:
:   ```
    <numpy.lib.index_tricks.IndexExpression object at 0x11543b0>
    ```

### nbytes

Value:
:   ```
    {<type 'numpy.int64'>: 8, <type 'numpy.int16'>: 2, <type 'numpy.comple
    x128'>: 16, <type 'numpy.uint64'>: 8, <type 'numpy.bool_'>: 1, <type '
    numpy.complex256'>: 32, <type 'numpy.float32'>: 4, <type 'numpy.int8'>
    : 1, <type 'numpy.uint8'>: 1, <type 'numpy.uint16'>: 2, <type 'numpy.o
    bject_'>: 4, <type 'numpy.float64'>: 8, <type 'numpy.int32'>: 4, <type
     'numpy.string_'>: 0, <type 'numpy.void'>: 0, <type 'numpy.float128'>:
     16, <type 'numpy.int32'>: 4, <type 'numpy.uint32'>: 4, <type 'numpy.u
    nicode_'>: 0, <type 'numpy.complex64'>: 8, <type 'numpy.uint32'>: 4}
    ```

### sctypeDict

Value:
:   ```
    {0: <type 'numpy.bool_'>,
     1: <type 'numpy.int8'>,
     2: <type 'numpy.uint8'>,
     3: <type 'numpy.int16'>,
     4: <type 'numpy.uint16'>,
     5: <type 'numpy.int32'>,
     6: <type 'numpy.uint32'>,
     7: <type 'numpy.int32'>,
    ...
    ```

### sctypeNA

Value:
:   ```
    {'?': 'Bool',
     'B': 'UInt8',
     'Bool': <type 'numpy.bool_'>,
     'Complex128': <type 'numpy.complex256'>,
     'Complex32': <type 'numpy.complex64'>,
     'Complex64': <type 'numpy.complex128'>,
     'D': 'Complex64',
     'F': 'Complex32',
    ...
    ```

### sctypes

Value:
:   ```
    {'complex': [<type 'numpy.complex64'>,
                 <type 'numpy.complex128'>,
                 <type 'numpy.complex256'>],
     'float': [<type 'numpy.float32'>,
               <type 'numpy.float64'>,
               <type 'numpy.float128'>],
     'int': [<type 'numpy.int8'>,
             <type 'numpy.int16'>,
    ...
    ```

### typeDict

Value:
:   ```
    {0: <type 'numpy.bool_'>,
     1: <type 'numpy.int8'>,
     2: <type 'numpy.uint8'>,
     3: <type 'numpy.int16'>,
     4: <type 'numpy.uint16'>,
     5: <type 'numpy.int32'>,
     6: <type 'numpy.uint32'>,
     7: <type 'numpy.int32'>,
    ...
    ```

### typeNA

Value:
:   ```
    {'?': 'Bool',
     'B': 'UInt8',
     'Bool': <type 'numpy.bool_'>,
     'Complex128': <type 'numpy.complex256'>,
     'Complex32': <type 'numpy.complex64'>,
     'Complex64': <type 'numpy.complex128'>,
     'D': 'Complex64',
     'F': 'Complex32',
    ...
    ```

### typecodes

Value:
:   ```
    {'All': '?bhilqpBHILQPfdgFDGSUVOMm',
     'AllFloat': 'fdgFDG',
     'AllInteger': 'bBhHiIlLqQpP',
     'Character': 'c',
     'Complex': 'FDG',
     'Datetime': 'Mm',
     'Float': 'fdg',
     'Integer': 'bhilqp',
    ...
    ```

  


| Home | Trees | Indices | Help | PyDSTool |
| --- | --- | --- | --- | --- |

|  |  |
| --- | --- |
| Generated by Epydoc 3.0.1 on Fri May 4 15:24:04 2012 | http://epydoc.sourceforge.net |
